# Antioxidant Properties of Probiotic Bacteria

**DOI:** 10.3390/nu9050521

**Published:** 2017-05-19

**Authors:** Yang Wang, Yanping Wu, Yuanyuan Wang, Han Xu, Xiaoqiang Mei, Dongyou Yu, Yibing Wang, Weifen Li

**Affiliations:** Key Laboratory of Molecular Animal Nutrition of the Ministry of Education, Institute of Feed Science, College of Animal Sciences, Zhejiang University, Hangzhou 310058, China; 11317017@zju.edu.cn (Y.W.); ypwu0902@163.com (Y.W.); yuanzizju@163.com (Y.W.); 18868107975@139.com (H.X.); 21617067@zju.edu.cn (X.M.); dyyu@zju.edu.cn (D.Y.)

**Keywords:** oxidative stress, antioxidant, probiotic, signaling pathway, gut microbiota

## Abstract

Oxidative stress defines a condition in which the prooxidant–antioxidant balance in the cell is disturbed, resulting in DNA hydroxylation, protein denaturation, lipid peroxidation, and apoptosis, ultimately compromising cells’ viability. Probiotics have been known for many beneficial health effects, and the consumption of probiotics alone or in food shows that strain-specific probiotics can present antioxidant activity and reduce damages caused by oxidation. However, the oxidation-resistant ability of probiotics, especially the underling mechanisms, is not properly understood. In this view, there is interest to figure out the antioxidant property of probiotics and summarize the mode of action of probiotic bacteria in antioxidation. Therefore, in the present paper, the antioxidant mechanisms of probiotics have been reviewed in terms of their ability to improve the antioxidant system and their ability to decrease radical generation. Since in recent years, oxidative stress has been associated with an altered gut microbiota, the effects of probiotics on intestinal flora composition are also elaborated.

## 1. Introduction

Oxidative stress refers to elevated intracellular levels of oxygen radicals that cause damage to lipids, proteins, and DNA [1]. Reactive oxygen species (ROS), including superoxide anion radicals, hydroxyl radicals, and hydrogen peroxide, are one of the highly active oxygen free radicals. During evolution, most living organisms possess enzymatic defenses (superoxide dismutase (SOD), glutathione peroxidase (GPx), glutathione reductase (GR)), non-enzymatic antioxidant defenses (glutathione, thioredoxin, Vitamin C, Vitamin E), and repair systems to protect them against oxidative stress [2]. However, these native antioxidant systems are generally not enough to prevent living organisms from oxidative damage. Antioxidant additives using substances that delay or prevent the oxidation of cellular substrates have demonstrated the capacity to protect the human body against oxidative damage. Although several synthetic antioxidants, including butylated hydroxyanisole and butylated hydroxytoluene, have been widely used in retarding lipid oxidation, their safety has recently been questioned due to liver damage and carcinogenicity [3]. Therefore, in recent years, finding safer and natural antioxidants from bio-resources to replace synthetic antioxidants has received a great deal of attention.

Recent studies have led to a renewed interest in probiotics, which are claimed to have health benefits. Probiotics refer to live nonpathogenic microorganisms, which, when administered in adequate amounts, confer microbial balance, particularly in the gastrointestinal tract [4]. Evidence has showed that probiotic bacteria present significant antioxidant abilities both in vivo and in vitro [5,6,7,8]. Hence, with the increasing popularity of probiotic bacteria, we set out the antioxidant properties of probiotic bacteria and underscore their mode of action in this review.

## 2. Reactive Oxygen Species 

**Figure 1:**
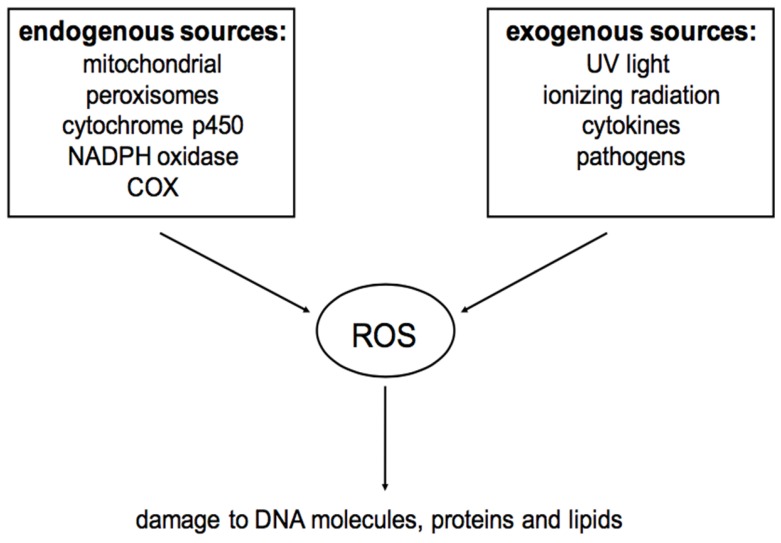
Sources of reactive oxygen species (ROS) (referred to [21]).

Except for anaerobic organisms, oxygen is essential for all animals and plants. Hypoxia occurs when the oxygen concentration is below the normal level, which will lead to injuries and even death. Conversely, oxidative stress will happen if the oxygen concentration is over the normal level. Many factors can force organisms to experience oxidative stress such as cigarettes, herbicides, nitrogen oxides, ozone, radiation, and some metal [9]. ROS, including oxygen ions and peroxides, are the products of normal oxygen consuming metabolic process and are often associated with the principle of oxidative stress [10]. ROS can be both endogenously and exogenously generated. The sources of ROS can be found in Figure 1. Due to their highly reactive nature, ROS can modify other oxygen species, DNA, proteins, or lipids. It is believed that excessive amounts of ROS can cause genomic instability [11], leading to a variety of chronic diseases, including atherosclerosis, arthritis, diabetes, alzheimer’s disease, neurodegenerative diseases, and cardiovascular diseases [12,13,14,15,16]. 

However, it is worth noting that, in addition to oxidative stress, when maintained at proper cellular concentrations, ROS are important for cell signaling roles [17]. In the past decades, researchers have found that ROS could serve as second messengers to regulate biological processes [18]. In 1991, transcription factors of the nuclear factor kappa-light-chain-enhancer of activated B cells of the (NFκB)/rel family were first unveiled to be activated by the induction of oxidizing agents or ionizing radiation [19]. Subsequently, some other transcription factors and kinases, including hypoxia-inducible factors (HIFs) and phosphatidylinositol 3-kinase (PI3K), have been discovered to possess redox-sensitive elements that can be regulated by ROS [1,20]. Thus, the two faces of ROS make it difficult to use antioxidants because antioxidants would influence the normal redox biology.

## 3. Probiotics and Their Roles in Antioxidation

Previous reviews suggested that probiotics could lower the frequency and duration of diarrhea; stimulate humoral and cellular immunity; prevent cancer; and decrease unfavorable metabolites, including ammonium and procancerogenic enzymes in the colon [2]. Moreover, in recent years, a great number of studies have focused on the impacts of intestinal microbiota on an individual’s health status. Probiotics, which are capable of colonizing the intestinal tract, are reported to improve metabolic diseases such as obesity and diabetes through modulating intestinal microorganisms [22,23,24]. 

Lactic acid bacteria (LAB) strains are the major representatives of probiotics both in the food and pharmaceutical market [25]. LAB were reported to have positive effects on the treatment and maintenance of ulcerative colitis (UC) [26,27,28]. They were also associated with the improvement of metabolic diseases [23,29]. Additionally, in fishes, *Lactobacillus rhamnosus* or/and *Lactobacillus lactis* played a beneficial role in improving the growth, immune system, and oxidative status of sea bream, *Pagrus major* [30,31]. Probiotic *Bifidobacterium* is also a very commonly used probiotic bacterium. It was able to promote antitumor immunity [32] and relieve irritable bowel syndrome in women [33]. *Bacillus* species are preferred in the feed industry because of their stability as spore-forming bacteria and ability to produce a variety of enzymes such as protease, amylase, and lipase [34]. The intestinal microbiota and mucosal immunity of fish could be shaped by *Bacillus* [35], and the mucosal immunity of chickens could also be improved via *Bacillus* treatment [36]. 

In addition to the beneficial effects mentioned above, in recent decades, many findings have shed new light on the understanding of the antioxidant capacity of probiotics. The culture supernatant, intact cells, and intracellular cell-free extracts of *Bifidobacterium animalis* 01 were found to scavenge hydroxyl radicals and superoxide anion in vitro while enhancing the antioxidase activities of mice in vivo [6]. Further, the oxidative stress in patients with type 2 diabetes can be ameliorated by multispecies probiotics [37]. LAB stains have been studied widely both in animals and the human body. It is revealed that LAB can resist ROS, including peroxide radicals [38], superoxide anions, and hydroxyl radicals [39]. Rats fed high-fat diets supplemented with *Lactobacillus plantarum* P-8 presented an elevated antioxidant ability, as reflected by curtailing the accumulation of liver lipids and protecting healthy liver function [40]. In humans, *Lactobacillus rhamnosus* exerted strong antioxidant activity in situations of elevated physical stress. Athletes exposed to oxidative stress might benefit from the ability of *Lactobacillus rhamnosus* to increase antioxidant levels and neutralize the effects of reactive oxygen species [41].

## 4. Modes of Action of Probiotic Bacteria in Antioxidation

**Figure 2:**
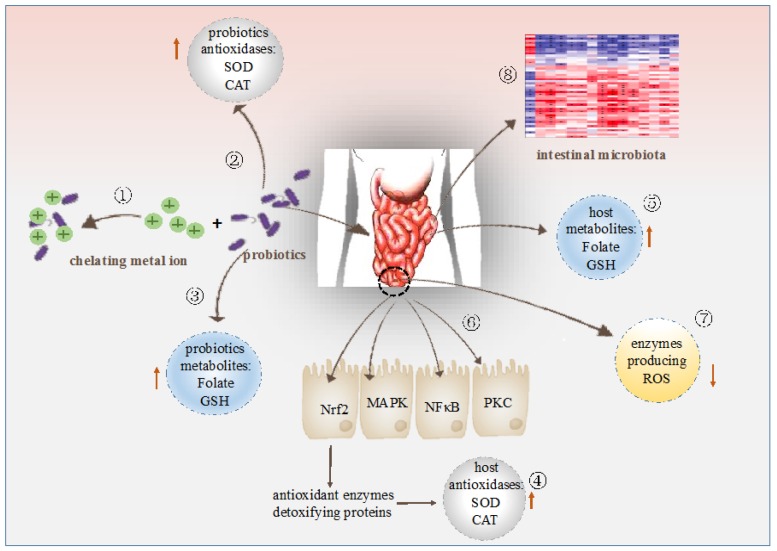
Modulation of antioxidation by probiotics. ① Probiotics chelate metal ion. ② Probiotics possess their own antioxidases. ③ Probiotics produce antioxidant metabolites. ④ Probiotics up-regulate antioxidase activities of the host. ⑤ Probiotics increase levels of antioxidant metabolites of the host. ⑥ Probiotics regulate signaling pathways. ⑦ Probiotics down-regulate activities of enzymes producing ROS. ⑧ Probiotics regulates intestinal microbiota.

During the past decades, studies have demonstrated that different probiotic bacteria strains could exert antioxidant capacity in different ways. However, few reviews regarding the basis for the antioxidant mechanisms of probiotics have been found. Thus, the following sections provide an overview of the existing knowledge on the oxygen resistant mechanisms of various probiotic strains (Figure 2).

### 4.1. Metal Ion Chelating Ability

Chelators, such as ethylene diamine tetraacetic acid (EDTA), bathophenanthrolinedisulfonic acid (BPS), penicillamine, and desferrioxamine, have been reported to capture metal ions and prevent metal ions from catalyzing the oxidation [42]. The metal ion (ferrous and cupric ions) chelating ability of 19 LAB strains was measured by Lin and Yen in 1999 [5]. The results showed that *Streptococcus thermophilus* 821 demonstrated the best chelating ability for both Fe^2+^ and Cu^2+^. Other strains also showed a chelating ability for either Fe^2+^ or Cu^2+^. In addition, another LAB strain, *Lactobacillus casei* KCTC 3260, was found to possess a high antioxidant ability by chelating Fe^2+^ or Cu^2+^, although no detectable SOD activity was observed [43]. Similarly, the intracellular cell-free extract of *Lactobacillus helveticus* CD6 also showed higher Fe^2+^ ion chelation [44]. Although the factors responsible for metal ion chelation are not well understood in probiotic bacteria, it is revealed that the transition metal ion can inhibit enzyme-catalyzed phosphate ester displacement reactions and produce peroxyl and alkoxyl radicals by the decomposition of hydroperoxides [45]. Hence, in the opinion of Lin and Yen, the chelating capacity of those probiotic strains may be due to the physiological chelators that exist in the intracellular cell-free extract of probiotics [5].

### 4.2. Antioxidant Enzymes System

Like animals, probiotics also have their own antioxidant enzymatic systems. One of the best known of these enzymes is SOD. Superoxide is one of the most abundant ROS produced by the mitochondria, while SOD catalyzes the breakdown of superoxide into hydrogen peroxide and water and is therefore a central regulator of ROS levels [46]. Bacteria can employ Fe-SOD and Mn-SOD, but mammals utilize both cytoplasmic and extracellular forms of Cu, Zn-SOD, and mitochondrial Mn-SOD, which is closely related to the bacterial Mn-SOD in evolutionary terms [47]. In the study of Kullisaar and colleagues, *Lactobacillus fermentum* E-3 and E-18 were able to express Mn-SOD to resist oxidative stress [39]. Although the antioxidant activity of SOD is well-known [48,49,50], the therapeutic application of SOD is limited, mainly because of its short circulatory half-life, which restricts its bioavailability. In order to address this problem, efforts have been made to find suitable vehicles for SOD. Probiotic bacteria capable of local delivery of SOD open a novel approach to bowel diseases characterized by ROS production. Recently, a study exploring the impact of the engineered *Lactobacillus casei* BL23 strains producing SOD on mice with Crohn's disease demonstrated that mice receiving engineered strains had a faster recovery of initial weight loss, increased enzymatic activities in the gut, and a lesser extent of intestinal inflammation than the control mice [51]. 

Catalase (CAT) participates in cellular antioxidant defense by decomposing hydrogen peroxide, thereby preventing the generation of hydroxyl radicals by the Fenton reaction [52]. LAB are generally CAT-negative [53]; however, de LeBlanc and colleagues proved that a CAT-producing *Lactococcus slactis* could prevent 1,2-dimethylhydrazine-induced colon cancer in mice. Additionally, engineered *Lactobacillus casei* BL23 strains producing CAT were able to prevent or decrease the severity of intestinal pathologies caused by ROS [54].

Moreover, probiotics can also stimulate the antioxidant system of the host and elevate the activities of antioxidases efficiently. Studies in pigs showed that dietary *Lactobacillus fermentum* supplementation could increase serum SOD and GPx and enhance hepatic CAT, muscle SOD, and Cu and Zn-SOD compared to the control group [55]. Additionally, the intake of yeast probiotic at different dosages augmented the body weight and GPx activity of chicks [56]. Consistent with this, research in humans have shown an increased erythrocyte SOD and GPx activities as well as total antioxidant status in type 2 diabetic patients receiving yogurt containing *Lactobacillus acidophilus* La5 and *Bifidobacterium lactis* Bb12 [57]. Furthermore, our previous in vitro research also implied that *Bacillus amyloliquefaciens* SC06 elevated CAT and GST gene expressions and the CAT activity in intestinal porcine epithelial cells-1 (IPEC-1) [7].

### 4.3. Antioxidant Metabolites

Probiotics can produce various metabolites with antioxidant activity, such as glutathione (GSH), butyrate, and folate. Folate is a vitamin that accepts one-carbon units from donor molecules and is involved in many metabolic pathways. The efficiency of DNA replication, repair, and methylation is affected by folate availability [58]. Due to potentially antioxidant applications, the ability to produce folate has been intensively investigated in multiple probiotic strains from a variety of origins [59]. Evidence showed that the folate-producing *Bifidobacteria* enhanced the folate status in both rats and human [60,61]. In addition, Ahire and colleagues reported that the intracellular cell-free extract of folate producing probiotic *Lactobacillus helveticus* CD6, demonstrated antioxidant potentials as the intact cell did [44]. GSH, a major cellular non enzymatic antioxidant, eliminates radicals like hydrogen peroxides, hydroxyl radicals, and peroxynitrite mainly via cooperation with selenium dependent glutathione peroxidase [62]. Kullisaar and colleagues found that the two antioxidative *Lactobacillus fermentum* strains, E-3 and E-18, contained remarkable levels of GSH [39]. Furthermore, for the first time, their research group found a whole GSH system existed in *Lactobacillus fermentum* ME-3 [63]. Butyrate is a short-chain fatty acid (SCFA) produced by microbiota in the colon and distal small intestine from resistant starch, dietary fiber, and low-digestible polysaccharides by fermentation [64]. The MIYAIRI 588 strain of *Clostridium butyricum* is a butyrate-producing probiotic. It has been recently shown to induce antioxidases in rats with nonalcoholic fatty liver disease to suppress hepatic oxidative stress [65]. 

The levels of antioxidant metabolites of the host can also be regulated by probiotics treatment. Folate and vitamin B12 deficiency promoted oxidative stress in adult type 2 diabetes [66]. In the study of Mohammad and colleagues, a randomized nutritional supplementation trial was performed in free-living children of both sexes. Daily consumption of the *Lactobacillus acidophilus* La1 yoghurt significantly improved the mean levels of plasma folate and vitamin B12 in the studied children compared with the respective baseline data [67], indicating an improved oxidative status. Vitamin B1 is able to rescue cells and animals from oxidative stress [68,69]. In healthy young women, daily consumption of 200 g of both probiotic and conventional yoghurt for two weeks contributed to the total intake of vitamin B1, which was reflected by increased levels of plasma thiamine [70]. Additionally, the GSH level and biosynthesis of GSH were also enhanced in rats treated with probiotics in order to reduce oxidative stress in experimental acute pancreatitis [71,72].

### 4.4. Antioxidant Signaling Pathway Mediated by Probiotic Bacteria

#### 4.4.1. Nrf2-Keap1-ARE

A well-studied system for transducing exogenous stimuli into eukaryotic transcriptional responses is the Nrf2-Keap1-ARE pathway. Nrf2 activation upregulates a series of genes including those involved in xenobiotic and ROS detoxification in order to resist oxidant and electrophilic environmental stressors [73,74]. At low levels of ROS, Nrf2 is bound to its cytoplasmic inhibitor Keap1, which suppresses the activity of Nrf2 by targeting it for constitutive polyubiquitination through a Cullin3-based E3 ligase complex and consequent proteasomal degradation [75]. Keap1 is considered a molecular switch of Nrf2. When cells are attacked by free radicals or nucleophiles, the redox-sensitive cysteine residues of Keap1 react and alter the functional conformation of Keap1, thereby abolishing the inactivation of Nrf2 [76]. Thereafter, Nrf2 translocates to the nucleus and binds to antioxidant response element (ARE) sequences, promoting the transcription of ARE-driven genes such as genes encoding antioxidant enzymes and detoxifying proteins [77,78,79]. In recent years, both in vivo and in vitro reports have indicated that probiotic bacteria could protect against oxidative stress through regulating the Nrf2-Keap1-ARE pathway. Recently, a high cholesterol diet (hyperlipidemic models, HM) has been employed to construct hyperlipidemic models of male Kunming mice. Hyperlipidemic and normal mice were then treated with *Lactobacillus plantarum* CAI6, *Lactobacillus plantarum* SC4, and physiological saline through oral gavage. As expected, the Nrf2 levels in the liver and kidneys were much higher in mice receiving HM. Moreover, compared with the HM group, liver Nrf2 was significantly increased in mice fed with the HM/CAI6 and HM/SC4 diets [80]. Similarly, the antioxidant and hypolipidemic effects of *Lactobacillus plantarum* FC225 were investigated in mice fed a high fat diet. The scavenging activities of superoxide anion radicals were enhanced by *Lactobacillus plantarum* FC225. The flow cytometric analysis of Nrf2 expression and translocation in the hepatocyte of *Lactobacillus plantarum* FC255-treated mice was markedly promoted, and *Lactobacillus plantarum* FC225 was further able to prevent a high fat diet-induced inhibition of antioxidant enzymes [81]. As mentioned, *Clostridium butyricum* MIYAIRI 588 is a butyrate-producing probiotic. Endo and colleagues established the rat nonalcoholic fatty liver disease model with a choline-deficient/l-amino acid (CDAA)-defined-diet [65]. According to their findings, treatment with MIYAIRI 588 elevated hepatic antioxidant enzyme activities in (CDAA)-defined-diet-induced rats via the activation of Nrf2 expressions [65]. Additionally, recent evidence obtained by our research group also demonstrated that probiotic *Bacillus amyloliquefaciens* SC06 ameliorated the H_2_O_2_-induced IPEC-1 oxidative stress by decreasing ROS levels and regulating Nrf2 expressions [7] (Table 1).

#### 4.4.2. NFκB

The first eukaryotic transcription factor shown to respond directly to oxidative stress was NFκB [19]. It is reported that, during inflammation, ROS can mediate the activation of redox-sensitive transcription factor NFκB, and the subsequent expression of inflammatory cytokines [82]. The extracellular polysaccharide from *Bacillus* sp. strain LBP32 prevented LPS-induced inflammation in RAW 264.7 macrophages by inhibiting NFκB and ROS production [83]. Moreover, probiotic mixture VSL#3 could inhibit NFκB and induce heat shock proteins in colonic epithelial cells [84] (Table 1).

#### 4.4.3. MAPK

Mitogen-activated protein kinases (MAPKs) includes four subfamilies, the best characterized of which are the extracellular regulated protein kinases (ERKs), c-jun N-terminal kinase (JNKs), and p38-MAPK; these can be activated by a variety of stimuli [85]. In general, ERKs are mainly involved in anabolic processes such as cell division, growth, and differentiation, whereas JNKs and p38-MAPK are mostly associated with cellular responses to diverse stresses such as UV irradiation and osmotic shock [86,87]. Soluble factors of conditioned media from the probiotic *Lactobacillus* GG (*Lactobacillus* GG-CM) could induce both heat shock protein (Hsp)25 and Hsp72 in a time- and concentration-dependent manner in young adult mouse colon (YAMC) cells. Tao and colleagues suggested that the pretreatment of cells with *Lactobacillus* GG-CM alone activated all three MAPKs investigated [88]. Exposure of YAMC cells to inhibitors against p38 and JNK before *Lactobacillus* GG-CM treatment resulted in the blockade of Hsp72 expression, which confirmed the role for MAPK signaling pathways in the induction of Hsps by *Lactobacillus* GG-CM in epithelial cells [88]. In addition, Seth and colleagues used the strong oxidizer H_2_O_2_ to induce the disruption of tight junctions and barrier function in Caco-2 cell monolayers [85]. In their study, *Lactobacillus rhamnosus* GG was observed to the produce soluble proteins p40 and p75, which were able to ameliorate the H_2_O_2_-induced epithelial barrier disruption by a MAPK-dependent mechanism [85] (Table 1).

#### 4.4.4. PKC

Protein kinase C (PKC) represents a family of phospholipid-dependent ser/thr kinases that are involved in a variety of pathways that regulate cell growth, death, and stress responsiveness [89,90]. Evidence demonstrates that PKC is among a group of cell-signaling molecules that are sensitive targets for redox modification [91]. As aforementioned, besides MAPKs, the attenuation of H_2_O_2_-induced redistribution of tight junction proteins by the aforementioned soluble proteins produced by *Lactobacillus rhamnosus* GG was abrogated by Ro-32-0432, a PKC inhibitor [85] (Table 1).

### 4.5. Regulation of The Enzymes Producing ROS

Oxidative stress is derived either from an increase in ROS production or decreased levels of ROS-scavenging proteins. Thus, recently many studies have investigated the effects of probiotics on ROS production. ROS are generated by several enzymatic reactions and chemical processes [92]. NADPH oxidase (NOX) complex is considered to be a major source of ROS generation [93,94,95]. It is now known that there are seven human NOX homologues (NOX1–5, dual oxidase 1 (DUOX1), and DUOX2) that function to purposely produce ROS for a range of host defense and signaling functions [96]. The catalytic subunit of this complex is NOX2. NOX2 does not generate superoxide on its own; rather, stimulating the neutrophil causes the recruitment of cytosolic factors that include p40^phox^, p47^phox^, p67^phox^, and the small GTPase RAC1. These cytosolic factors combine with the membrane-bound NOX2 and p22^phox^ to generate the classic phagocyte response to stimulation known as the respiratory burst [97,98]. Recently, Gómez-Guzmán and colleagues have suggested that probiotics *Lactobacillus fermentum* CECT5716 and *Lactobacillus coryniformis* CECT5711 (K8) plus *Lactobacillus gasseri* CECT5714 (LC9) (1:1) were able to decrease NOX activity and mRNA expressions of NOX-1 as well as NOX-4 in spontaneously hypertensive rats [99]. The oxidative stress of H_2_O_2_-induced IPEC-1 was also shown as decreased NOX activity and p47^phox^ expression by *Bacillus amyloliquefaciens* SC06 treatment [7]. 

Cyclo-oxygenase (COX) is a rate-limiting enzyme in prostaglandin biosynthesis and a two-step enzymatic process in which ROS are generated [100]. COX-2 was upregulated in atherosclerotic lesions [101] and catalyzes the production of the majority of vascular prostanoids in human atherosclerotic areas [102]. Down-regulated COX-2 was found in helicobacter pylori-infected mongolian gerbils with a commercial probiotic Lacidofil treatment [103]. Patel and colleagues demonstrated that *Lactobacillus acidophilus* pretreatment decreased COX-2 expression in catla thymus macrophages compared to *Aeromonas hydrophila* and co-stimulated macrophages [104]. 

The cytochrome P450 (CYP) enzymes are important in the oxidative metabolism of xenobiotic agents [105]. Poor coupling of the P450 catalytic cycle leads to continuous production of ROS, which affects signaling pathways and other cellular functions [106]. A few studies have explored the role of probiotics in regulating CYP. With the application of *Lactobacillus casei*, the expression of the CYP1A1 enzyme was found to be decreased in the proximal part of the jejunum and colon of male rats. Meanwhile, the CYP1A1 mRNA level was also decreased in the distal part of the jejunum, ileum, and caecum [107]. In the study by Sharan, the effects of milk, Dhi, and four probiotic Dahi preparations, namely Acidophilus Dahi, Plantarum Dahi, Acidopholus-plantarum Dahi, and Acidophilus-bifidus Dahi, on CYP and the antioxidase activities of rats was investigated [108]. Feeding Acidophilus Dahi for eight weeks could up-regulate the activities of quinone reductase and glutathione S-transferase in the liver and glutathione S-transferase in the colon. Accompanying this, the activities of CYP1A1, CYP1A2, and CYP1B1 were significantly decreased in the livers of rats fed Acidopholus-plantarum Dahi and Acidopholus-plantarum Dahi or Acidopholus-plantarum Dahi and Acidophilus-bifidus [108].

### 4.6. Regulation of The Intestinal Microbiota

#### 4.6.1. The Intestinal Microbiota Composition

Infants develop their microbiome at or before birth and by exposure to bacteria through the birth canal or contact with maternal skin in the case of Caesarian born babies [109]. Gut colonization of the newborn begins with facultative anaerobes such as enterobacteria and streptococci and continues with anaerobic genera, including *Bifidobacterium*, *Bacteroides*, and *Clostridium* [110,111]. The gut microbial composition in the newborn undergoes substantial modulation with a number of environmental and genetic factors to ultimately form the commensal intestinal microbiota. Each human adult harbors approximately 10^14^ bacteria in the gut, which is about 10 times the number of cells making up the human body [112]. There are at least 400–500 different bacterial species, which can be divided into different strains, highlighting the enormous complexity of this ecosystem [113]. The majority of bacterial species in a healthy human gut is *Bacteroidetes* (including *Clostridia* and *Bacilli*) and *Firmicutes* (*Bacteroides fragilis* and *Bacteroides thetaiotaomicron*) [114]. In contrast, *Proteobacteria*, *Actinobacteria*, *Fusobacteria*, *Cyanobacteria*, and *Verrucomicrobia* are less abundant phyla [115]. Moreover, different bacterial groups are enriched at different sites. It is reported that the *Bacilli* class of the *Firmicutes* and *Actinobacteria* is rich in the small intestine, whereas the *Bacteroidetes* and *Lachnospiraceae* families of the *Firmicutes* were more prevalent in the colon [116]. In addition, microbiota composition in the intestinal lumen differs significantly from the microbiota attached and embedded in the mucus layer as well as the microbiota in the immediate proximity of the epithelium [117].

#### 4.6.2. The Antioxidant Influences of Probiotics by Regulating Microbiota Composition

Gut microbiota protects its host from pathogens by competitive exclusion, including occupation of attachment sites, consumption of nutrient sources, and production of antimicrobial substances [117]. When the intestinal microbiota is abnormal, harmful bacteria will proliferate excessively, inducing the endotoxin in blood and causing significant oxidative stress. Microbial contact-induced epithelial ROS generation is an extremely conserved phenomenon across phyla. This mechanism is a general mean by which bacterial communities can affect redox homeostatic in the host [118]. Although the functions of probiotics in altering intestinal microbiota composition and gut diseases have been reviewed [119,120], there have been relatively few studies that rigorously characterize the effect of probiotics on antioxidation regarding the intestinal microbiota composition. Probiotics are regular habitants of the gastrointestinal tract of both humans and animals [121,122], and they can regulate the composition of intestinal microbiota and inhibit the excessive proliferation of harmful bacteria, which may contribute to decreased oxidative stress. *Lactobacillus* and *Bifidobacterium*, the most common probiotic species, producing lactic acid, acetic acid, and propionic acid, can lower the intestinal pH and suppress the growth of various pathogenic bacteria to keep the balance of the gut flora [123,124]. *Lactobacillus rhamnosus* GG, which has been shown to secrete a low-molecular-weight compound, can inhibit a broad spectrum of gram-positive, gram-negative, and anaerobic bacteria [125]. Additionally, for specific probiotic strains, the production of various substances such as organic acids, bacteriocins, and biosurfactants, are toxic to pathogenic microorganisms [126]. Moreover, gut microbes influence the metabolism of cells in tissues outside of the intestines, such as liver and adipose tissue, and thereby modulate lipid and glucose homeostasis, as well as systemic inflammation, in the host [127]. Recently, Qiao and colleagues have reported that the alterations of gut microbiota in high-fat diet (HFD)-fed mice was strongly linked to oxidative stress [128]. The changes in intestinal microbiota following HFD could promote metabolic endotoxemia and trigger metabolic disorders, including obesity and oxidative stress [127]. According to the research of Xin and colleagues, *Lactobacillus johnsonii* BS15 attenuated the HFD-induced oxidative stress of mice and change the *Firmicutes*/*Bacteroidetes* of gut microbiota [129]. Furthermore, supplementation of *Lactobacillus curvatus* HY7601 and *Lactobacillus plantarum* KY1032 in HFD-fed obese mice also induced gut microbial changes [130]. Everard and colleagues found that *Saccharomyces boulardii* reduce inflammation and fat mass in obese and type 2 diabetic mice. The effects of *Saccharomyces boulardii* on the host metabolism were associated with the dramatic changes in the gut microbial composition [131]. In a very recent study, in parallel with the improved intestinal endotoxemia, probiotic supplementation consisting of *Bifidobacterium infantis*, *Lactobacillus acidopilus*, and *Bacillus cereus*, increased the levels of these anaerobic bacteria but decreased the abundance of *Escherichia coli* and *Enterococcus* in the fecal sample of rats fed a high-sugar and high-fat diet [132]. 

## 5. Conclusions

The past few years have witnessed a tremendous growth in our knowledge concerning the beneficial effects of probiotics, especially those important in mediating responses to oxidative stress. However, the mechanisms of antioxidant action have not been properly understood. In this review, we summarize that probiotics may modulate the redox status of the host via their metal ion chelating ability, antioxidant systems, regulating signaling pathways, enzyme producing ROS, and intestinal microbiota. However, there are still many unsolved questions. It is controversial whether those in vitro results from animal experiments are transferable to humans. As most of the probiotics are incapable of colonizing the gut and are eliminated shortly after consumption [133], it is not clear what would be the outcome of prolonged administration. Thus, the complete picture of the interaction between probiotics and antioxidant capacity should be further investigated and come into view in the near future.

## Figures and Tables

**Table 1:** Summary of antioxidant signaling pathways mediated by probiotic bacteria.

Species	Signaling Pathway	Host	References
*Bacillus amyloliquefaciens* SC06	Nrf2-Keap1-ARE	IPEC-1 cell line	[7]
*Clostridium butyricum* MIYAIRI 588		rats	[65]
*Lactobacillus Plantarum* CAI6, SC4		mice	[80]
*Lactobacillus Plantarum* FC255		mice	[81]
*Bacillus* LBP32	NFκB	RAW 264.7 macrophages	[83]
VSL#3		colonic epithelial cells	[84]
*Lactobacillus rhamnosus* GG	MAPK	Caco-2 cell line	[85]
*Lactobacillus* GG-CM		YAMC cell line	[88]
*Lactobacillus rhamnosus* GG	PKC	Caco-2 cell line	[85]
